# Anti-Phosphatidylserine/Prothrombin Antibodies in Healthy Women with Unexplained Recurrent Pregnancy Loss

**DOI:** 10.3390/jcm10102094

**Published:** 2021-05-13

**Authors:** Daniel E. Pleguezuelo, Oscar Cabrera-Marante, Magdalena Abad, Edgard Alfonso Rodriguez-Frias, Laura Naranjo, Alicia Vazquez, Olga Villar, Francisco Javier Gil-Etayo, Manuel Serrano, Alfredo Perez-Rivilla, Laura de la Fuente-Bitaine, Antonio Serrano

**Affiliations:** 1 Department of Immunology, Hospital Universitario 12 de Octubre, 28041 Madrid, Spain; oscar.cabrera@salud.madrid.org (O.C.-M.); edgardalfonso.rodriguezde@salud.madrid.org (E.A.R.-F.); lauranaranjo92@gmail.com (L.N.); fgile@salud.madrid.org (F.J.G.-E.); aserrano@h12o.es (A.S.); 2 Department of Obstetrics and Gynecology, Hospital Universitario 12 de Octubre, 28041 Madrid, Spain; mabadgran@gmail.com (M.A.); aliciavazsaran@gmail.com (A.V.); olga.villar@salud.madrid.org (O.V.); lauradlfb@gmail.com (L.d.l.F.-B.); 3 Healthcare Research Institute, Hospital Universitario 12 de Octubre, 28041 Madrid, Spain; mserranobl@gmail.com; 4 Department of Microbiology, Hospital Universitario 12 de Octubre, 28041 Madrid, Spain; alfredo.perez@salud.madrid.org

**Keywords:** recurrent pregnancy loss, miscarriage, antiphospholipid syndrome, reproductive immunology, anti-phosphatidylserine/prothrombin, anticardiolipin, anti-beta-2-glycoprotein-I, lupus anticoagulant, heparin, hydroxychloroquine

## Abstract

Recurrent pregnancy loss (RPL) affects up to 6% of couples. Although chromosomal aberrations of the embryos are considered the leading cause, 50% of cases remain unexplained. Antiphospholipid Syndrome is a known cause in a few cases. Antiphospholipid antibodies (aPL) anticardiolipin, anti-Beta-2-Glycoprotein-I and Lupus Anticoagulant (criteria aPL) are recommended studies in RPL workup. We tested healthy women with unexplained RPL for criteria aPL and anti-Phosphatidylserine/Prothrombin antibodies (aPS/PT). Patients were classified into three groups according to the number and pregnancy week of RPL: Extra-Criteria (EC), with 2 miscarriages, Early Miscarriage (EM), with ≥3 before pregnancy at week 10 and Fetal Loss (FL), with ≥1 fetal death from pregnancy at week 10. Circulating criteria aPL were absent in 98.1% of EM, 90.9% of FL and 96.6% of EC groups. In contrast, aPS/PT were positive in 15.4% of EM, 15.1% of FL, 16.6% of EC patients and 2.9% in controls. aPS/PT posed a risk for RPL, with an odds ratio of 5.96 (95% confidence interval (CI): 1.85–19.13. *p* = 0.002) for EM, 7.28 (95% CI: 2.07–25.56. *p* = 0.002) for FL and 6.56. (95% CI: 1.77–24.29. *p* = 0.004) for EC. A successful live birth was achieved in all pregnant patients positive for aPS/PT who received treatment with heparin, aspirin and/or hydroxychloroquine.

## 1. Introduction

It is estimated that up to 6% of women suffer from recurrent pregnancy loss (RPL) [1,2,3]. The World Health Organization defined this condition in 1976 [4] as three or more consecutive pregnancy losses before the 20th week of pregnancy. The European Society for Human Reproduction and Embryology (ESHRE) recently updated this definition as the loss of two or more pregnancies [5]. The factors influencing such reproductive failure are usually found in 50% of cases [6,7], and include advanced age of the parents, genetic abnormalities of the products of conception [1], chronic endometritis, endometrial polyps, uterine synechiae, uterine anomalies, myomas, hydrosalpinx, endocrinological disorders, thyroid abnormalities, thrombophilia, sperm quality and life-style issues [8]. Within these causes, sporadic chromosomal anomalies in the embryos affect 60% of early pregnancy losses, mostly due to trisomies related to ageing parents [7]. Antiphospholipid Syndrome (APS) is estimated to affect 5% to 20% of women with recurrent pregnancy loss [7,9], with differing prevalence found in the literature according to the variable degree of morbidity of the population under study. For example, in Systemic Lupus Erythematosus (SLE), the prevalence of aPL increases up to 40% of patients [10]. 

APS is a systemic autoimmune disease which was first described in 1983 and characterized by arterial and venous thrombosis and obstetric morbidity [11,12]. It could present alone (primary), associated with other autoimmune diseases (SLE mainly) or in its catastrophic form (an acute and life-threatening condition with widespread thrombosis [13]). To date, there are no specific diagnostic criteria for APS. In order to identify patients with APS for research purposes, classification criteria (clinical and laboratory criteria) were established in Sapporo in 1998. The last revision of these classification criteria was carried out in Sydney in 2004 (published in 2006) [14]. These criteria are often used in clinical practice to describe patients with APS, but many do not fulfill the research-oriented definition due to their low sensitivity [15]. The classification criteria state that a patient with APS must meet at least one laboratory and one clinical criterion. Laboratory criteria include the presence of persistent antiphospholipid antibodies (aPL) such as lupus anticoagulant (LA), anticardiolipin (aCL) IgG/IgM or anti-beta-2-glycoprotein-I (aB2GPI) IgG/IgM [14]. Clinical criteria include vascular thrombosis (one or more clinical episodes of arterial, venous, or small vessel thrombosis, in any tissue or organ) and/or pregnancy morbidity (three or more consecutive miscarriages before week 10 of pregnancy when hormonal, anatomical or chromosomal aberrations are excluded; one pregnancy loss of a morphologically normal fetus beyond week 10; and premature birth before week 34 due to severe pre-eclampsia, eclampsia or placental insufficiency) [14].

It has been shown that aPL not only induce the formation of thrombi in arterial or venous vessels, but also affect placentation by interfering with trophoblast differentiation, extravillous cytotrophoblast invasion into the decidua [16] and syncytiotrophoblast apoptosis [17].

As a significant number of patients fulfilling the clinical criteria for APS do not carry detectable aPL included in the latest classification criteria (seronegative APS), an effort has arisen in the last decade to discover new aPL with clinical implications. Among these aPL, anti-Phosphatidylserine/Prothrombin antibodies (aPS/PT) were described in 2000 by Tatsuya Atsumi, Laura Bertolaccini and others [18]. These aPL, which were not included in the latest classification criteria set up in Sydney in 2004 [14], target the coagulation factor prothrombin when attached to the phospholipid phosphatidylserine [18]. These aPS/PT have been associated with many clinical manifestations of APS, such as thrombosis, ischemic cerebral events [19] and adverse pregnancy outcomes [20].

This study aimed to study whether aPS/PT are relevant or commonly found in patients with RPL.

## 2. Materials and Methods

This is a case–control study with the inclusion of patients from 2016 to 2018. Clinical data and serum samples were collected and analyzed from 2016 to 2020, with a follow-up time of at least two years for each patient.

Patients were evaluated according to the ESHRE protocol for RPL after their latest pregnancy loss in the reproduction unit of our Center for gynecological (endometritis, myomas, hydrosalpinx), anatomical (endometrial polyps, uterine synechiae, malformations), genetic (abnormal karyotypes of progenitors or the product of conception) or endocrinological causes, obesity, hypertension, cardiac and renal disease. A total of 115 women without the previously mentioned causes for RPL were referred to the Immunology Clinic for further studies.

### 2.1. Laboratory Studies

Patients were studied for the presence of aPL, in accordance with the Sydney criteria: anticardiolipin (aCL) IgG and IgM, anti-beta-2-glycoprotein-I (aB2GPI) of IgG and IgM isotypes, and lupus anticoagulant (LA). aCL and aB2GPI IgG/IgM were evaluated using a Bioplex-2200 system (Bio-Rad, Hercules, CA, USA) with our laboratory cutoffs (99th percentile). LA was measured using two methods, HemosIL dRVVT (cutoff ratio 1.20) and HemosIL Silica Clotting Time (cutoff ratio 1.30) assays (Instrumentation Laboratory SpA, Milano, Italy). LA was performed in all patients without active anticoagulation therapy. Patients positive for aPL were studied for the presence of antinuclear antibodies (ANA) and anti-double stranded DNA (anti-dsDNA). ANA were tested by indirect immunofluorescence (IIF) on Hep2 cells and anti-dsDNA were studied using the Crithidia lucilae immunofluorescence test.

The QUANTA Lite ELISA (INOVA DIAGNOSTICS, San Diego, CA, USA) was used to evaluate aPS/PT IgG & IgM. We had previously described our calculated aPS/PT for our local population based on the 99th percentile, which resulted in 30 U/mL for IgG and 40 U/mL for IgM [21].

### 2.2. Patients and Groups

**Figure 1:**
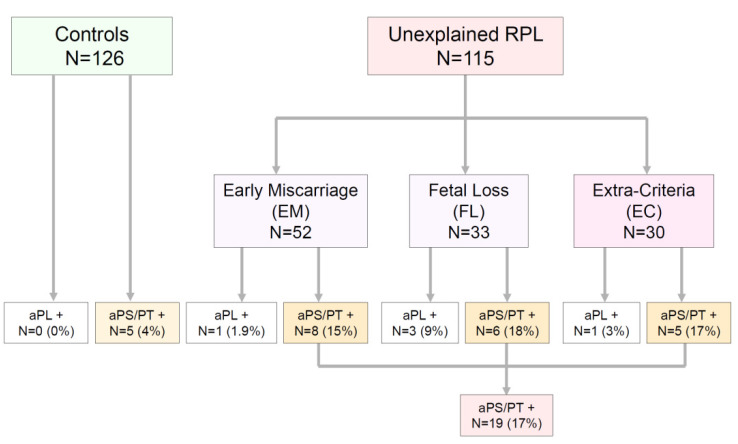
Positivities of criteria aPL (aPL) and aPS/PT among groups of patients and controls are shown. While positivity of criteria aPL did not surpass 10% in each one of the groups, aPS/PT was positive in 15–18% of patients.

As seen in Figure 1, 85 of the 115 patients included in the study met the Sydney classification criteria for APS, and 30 did not. Patients who met the clinical criteria were divided into two major groups according to the pregnancy week at which the pregnancy loss took place. Fifty-two women (45.2%) who had three or more consecutive and unexplained miscarriages before pregnancy week 10 were included in the group called ‘Early Miscarriage’ (EM). Thirty-three patients (28.7%) who had one or more deaths of a morphologically normal fetus from pregnancy week 10 were included in the group called ‘Fetal Loss’ (FL). To facilitate the interpretation of the results, patients who had a history of miscarriages before pregnancy week 10, but also experienced some fetal deaths, were excluded.

As aPL are part of the ESHRE recommended studies for women with two consecutive and unexplained miscarriages before pregnancy week 10 [5], an additional group of 30 women (26%) who did not fulfill the Sydney classification criteria for APS (‘Extra-Criteria’ or EC) was formed.

The control group consisted of 126 patient-age-matched healthy women who had an uncomplicated pregnancy/labor and who delivered an alive newborn in 2017 in our Center.

### 2.3. Follow-Up and Treatment

Patients with positive aPL results were followed-up and treated with subcutaneous low molecular weight heparin (LMWH) in prophylactic doses (enoxaparin 40 mg/day or tinzaparin 4500 U/day) and acetylsalicylic acid (ASA) at 100 mg/day orally from the day of ovulation until the end of the cycle. It was maintained during pregnancy and puerperium, with the dose of LMWH adjusted to match an anti-Xa between 0.3 and 0.5 throughout the pregnancy. It was stopped during labor and was restarted after delivery for an additional period of six weeks. ASA was continued until pregnancy week 37. In five women positive for aPL previously treated with LMWH and ASA without success, Hydroxychloroquine (HCQ) was added to LMWH and ASA, starting preconceptionally at 200 mg/day orally. After a positive pregnancy test, it was increased to 200 mg/12 h orally. APS-related possible pregnancy morbidities such as miscarriage, fetal death, pre-eclampsia, intrauterine growth restriction, prematurity or stillbirth were registered during follow-up.

### 2.4. Ethics

This study adhered to the Declaration of Helsinki and received a favorable report from the Institutional Review Board (ECCR) of Hospital Universitario 12 de Octubre (Reference Numbers 18/182 and 13/405). Informed consent was obtained from all subjects involved in the study. Clinical and analytical data were generated during the study and were stored in an anonymized database linked through a blind code.

### 2.5. Statistics

Qualitative variables were expressed as absolute values or percentages. Their associations were evaluated using Chi-Square or Fisher’s exact tests. The relative measure of effects was expressed as the odds ratio. Continuous variables were expressed as medians with interquartile range (IQR), and the Mann–Whitney-U test was used for their comparison. Odds ratio calculations and Multivariate analyses were performed using a logistic regression model. Probabilities under 0.05 were considered significant. Data were analyzed using MedCalc version 18.9 (MedCalc Software, Ostend, Belgium).

## 3. Results

### 3.1. Demographics

As seen in Table 1, there were no statistically significant differences in age within the patient groups (median: 37, 95% confidence interval (CI): 35–38) or between the age of patients compared to the controls (median: 37, 95% CI: 35–38; *p* = 0.510). Although the more prevalent ethnicity of the participants in this study was white-Caucasic, the population gathered as controls was more diverse than the groups of patients. BMI and the prevalence of overweight (measured before pregnancy) were higher in healthy controls than in patients. Regarding previously treated patients, only 4.4% of patients received APS-related treatments such as LMWH and ASA before aPS/PT testing. None were treated with HCQ.

### 3.2. Levels and Prevalence of aPL in Patients and Controls

Median levels of criteria aPL resulted in non-statistically significant differences, as interquartile ranges overlapped among groups (See Table 2). Levels of aPS/PT IgG did not differ significantly between controls and the Early Miscarriage (EM) group (*p* = 0.122) or between controls and the Fetal Loss (FL) group (*p* = 0.918). Controls and the Extra-Criteria (EC) group displayed slightly different but significant median aPS/PT IgG levels (*p* = 0.009). Levels of aPS/PT IgM were similar between controls and FL (*p* = 0.054), and between controls and the EC group (*p* = 0.127).

**Figure 2:**
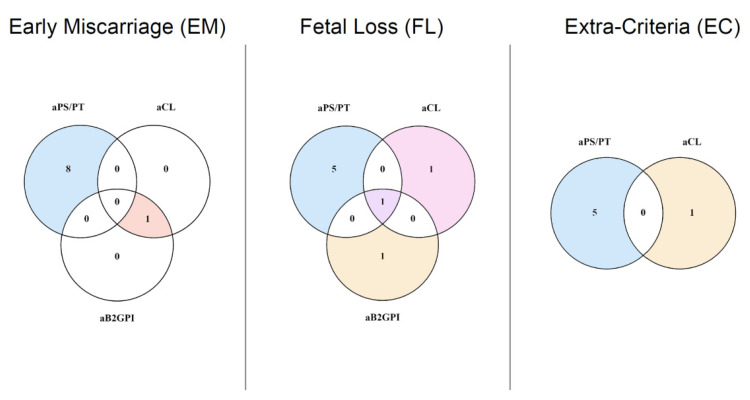
Venn diagrams of overlapping and isolated aPS/PT over criteria aPL. The number of women positive for aPS/PT, aCL and aB2GPI in each one of the groups in which patients were categorized is shown in this figure. LA is absent from this figure because none of our patients resulted in positive values. aPS/PT was mainly found in patients negative for criteria aPL.

As shown in Figure 1, in the EM group, only one patient (1.9%) showed positive values for criteria aPL, whereas 51 (98.1%) were negative. Among the negative results, eight (16% of seronegative patients and 15.4% of the total EM group) had positive values for aPS/PT. In the FL group, three patients (9.1%) had positive criteria aPL, while 30 (90.9%) were negative. Among this latter group, five (16.6% of seronegative patients and 15.1% of the total FL group) resulted in positive values of aPS/PT. Only one patient in the group composed of 30 patients not fulfilling the Sydney criteria had a positive aCL IgM, whereas 29 were negative for any criteria aPL. Five of them (17.2%) had circulating aPS/PT. None of the controls had positive criteria aPL and five (2.9%) resulted in positive levels of aPS/PT. aPS/PT and overlapping aPL among patients are depicted in Figure 2.

### 3.3. Univariate Analysis of aPS/PT Risk for RPL

Positive aPS/PT resulted in a significant risk for EM (odds ratio (OR): 5.96. 95% CI: 1.85–19.13. *p* = 0.002), for FL (OR: 7.28. 95% CI: 2.07–25.56. *p* = 0.002) and for EC (OR: 6.56. 95% CI: 1.77–24.29. *p* = 0.004) at the univariate analysis.

### 3.4. Multivariate Analysis of aPS/PT and Criteria aPL Risk for RPL

A multivariate logistic regression analysis was performed to compare the risk posed by criteria aPL versus aPS/PT in our cohorts of patients. Women carrying positive aPS/PT had a greater risk for EM (OR: 6.24. 95% CI: 1.94–20.08. *p* = 0.002), while criteria aPL did not result in a statistically significant contribution (*p* = 0.998). A similar result was found for FL, with aPS/PT displaying a significant risk (OR: 6.56. 95% CI: 1.77–24.29. *p* = 0.004), and criteria aPL failing to pose a risk for the outcome (*p* = 0.997). Another multivariate analysis showed that positive aPS/PT posed a risk for miscarriage in the EC group (OR: 6.83. 95% CI: 1.84–25.36. *p* = 0.004), whereas criteria aPL did not contribute to the outcome (*p* = 0.997).

‘A further multivariate logistic regression analysis (see Table 3) explored the risk posed by aPS/PT, criteria aPL, age and known cardiovascular risk factors, such as obesity and smoking habit. In the EM group, only aPS/PT (OR: 4.44. 95% CI: 1.34–14.70. *p* = 0.014) resulted in a significant contribution for the outcome. In the FL group, aPS/PT (OR: 5.68. 95% CI: 1.54–20.88. *p* = 0.008) showed a statistical association with fetal loss. In the EC group, both obesity and aPS/PT were observed to influence the outcome. Whereas aPS/PT were shown to pose a risk (OR: 4.51. 95% CI; 1.14–17.73. *p* = 0.031), obesity performed a protective role in our cohorts (OR: 0.17. 95% CI; 0.03–0.78. *p* = 0.022). The differences in BMI were investigated between the EC group and controls, and we found a lower median in patients (24.71. 95% CI: 22.36–26.82) than in healthy controls (27.06. 95% CI: 26.35–28.01).

### 3.5. Response to Treatment and Patient Follow-Up

Table 4 shows the results, treatment and outcome after the intervention for all patients who tested positive for any of the studied aPL. Four patients with positive aPL received treatment in the EM group. Among them, three carrying aPS/PT IgG achieved a new and uncomplicated pregnancy, with the delivery of a healthy newborn. The remaining patient had positive aCL and aB2GPI of IgM isotype that resulted in a new miscarriage, despite targeted treatment. Four patients received treatment in the FL group. Two carried aPS/PT IgM, one aCL IgM and another aB2GPI IgG antibodies. All of them had a successful new pregnancy. There were four treated patients in the EC group who were positive for at least one of the aPL studied. Three of them carried aPS/PT IgG and one was positive for aCL IgM. All four had successful new pregnancies after treatment. None of the patients who achieved a new pregnancy experienced any of the APS-related obstetrical morbidities such as pre-eclampsia, intrauterine growth restriction, prematurity or stillbirth.

## 4. Discussion

In our cohort composed of infertile but otherwise healthy women with RPL, we found that aPS/PT antibodies were more prevalent than aCL, aB2GPI and LA. Furthermore, these antibodies were mostly detected in women negative for other aPL. Both univariate and multivariate logistic regression analyses compared to criteria aPL recognized aPS/PT as significant risk factors for RPL, regardless at which week of pregnancy the loss happened. A good response to treatment was observed for all women who were positive for aPS/PT, thus giving strength to the possible involvement of these antibodies in the pathogenesis of some women with RPL. Secondly, we found that the prevalence, the statistical association and the response to the treatment of women with RPL carrying aPS/PT were similar in patients with two and three or more consecutive and unexplained miscarriages before pregnancy week 10. The concept of recurrent miscarriages is a matter of debate, with many groups considering a less rigorous definition [22]. We think our findings could reinforce the current recommendation for aPL testing after two miscarriages made by the ESHRE. This would help avoid the requirement of having a third consecutive miscarriage, which is required by the current classification criteria for APS [14].

It needs to be mentioned that we designed our study to exclude patients with a history of thrombosis, or those who had a diagnosis or symptoms related to other systemic autoimmune diseases such as SLE. We think this exclusion criteria could explain why none of our patients had positive LA, which is associated with a high risk of thrombosis. We think that the characteristics of the included patients show a greater resemblance to the average patient seen in the reproductive medicine units than those with complex diseases with dysregulated immune responses frequently evaluated in immunology clinics. However, this strict patient selection could bias our results and contribute to the low prevalence of aB2GPI, aCL and LA, given that the population tested was infertile but otherwise healthy. Another explanation for the low frequency of criteria aPL among our cohorts of patients could be the absence of a clear role of these antibodies in the pathogenesis of early miscarriages [23]. This has been reported and discussed earlier by Branch et al. [24] and by Clark et al. [23], who suggested that the mechanisms of aPL-mediated early miscarriages might be different from those causing late pregnancy loss or placental infarction.

Since aPS/PT have been proposed as a surrogate marker for LA in many research studies, it is remarkable that we found patients who tested positive for aPS/PT and negative for LA. Our results differ highly from the previously described correlation between aPS/PT and LA [25,26]. These conflicting results could be explained by the nature of patients included in our cohort, composed of infertile but otherwise healthy women and without diagnosis of other systemic autoimmune diseases or thrombosis episodes. However, we [27] and others [28,29] have previously commented on the discrepancy of aPS/PT and LA results in different cohorts of patients.

More work needs to be performed to address the pathogenicity of these aPS/PT and their correlation, or lack thereof, with LA in women with only obstetric manifestations of APS. An interesting article on the identification of different populations of aPS/PT was published recently by Chinnaraj and coworkers. They described that aPS/PT are a family of different antibodies all targeting phosphatidylserine-prothrombin complex but with different binding epitopes [30]. Depending on the epitope they target, some aPS/PT can bind to open or closed forms of prothrombin [30]. As far as we know, this type of study has not yet been carried out in obstetric APS patients, but it would help to elucidate why some women with isolated aPS/PT develop miscarriages and others do not, and why some women develop miscarriages earlier than others.

Testing of aPS/PT has increased since it was described in 2000 [18], with many groups showing the potential value of these aPL, which currently fall outside the Sydney classification criteria [14]. A systematic review of their association with the clinical manifestations of APS was published recently by Radin et al. [31]. In the pregnancy morbidity setting, our results are in line with those achieved by Zigon et al., who found a similar prevalence of aPS/PT in women with RPL [20]. In contrast to this study, Zigon et al. included, among women with RPL, 6% of patients with a history of thrombosis. Other groups have also tested aPS/PT in their patients with obstetrics manifestations. Among them, Mekinian et al. published a paper using the same aPS/PT detection kit but broader inclusion criteria. Their patients included women with at least three miscarriages before week 10, but also women with fetal loss, pre-eclampsia and prematurity due to placental insufficiency. In their study, 46% of patients presented concomitantly aPS/PT and other criteria aPL or LA, while isolated positives accounted only for 4.7% [32]. Other groups recently tested other Extra-Criteria aPL, with promising results such as anti-phosphatidylethanolamine antibodies [33,34].

Regarding treatment strategies and outcomes, previous works and a recent meta-analysis [35] have shown that LMWH in addition to ASA have improved the rate of successful pregnancies in women with obstetrical APS up to 80–85% [36]. Conversely, this benefit was not found in women with inherited thrombophilia, such as factor V Leyden, protein C, protein S or antithrombin deficiency [37]. Despite the progress achieved with LMWH and ASA in obstetric APS, there is still a 15–20% of cases refractory with this combination therapy [38,39]. The addition of HCQ, a drug possibly capable of restoring some of the damage exerted by aPL on trophoblasts in mice models [40,41], has been regarded as the next step in obstetrical APS treatment [42]. This drug has been reported to be safe in pregnancy and in women with obstetric APS pregnancies, with no [43,44] or minor side-effects [45], probably due to a limited duration of treatment [46]. However, we decided to be cautious and only prescribe HCQ to patients with a previously unsuccessful treatment regimen with LMWH and ASA. Moreover, HCQ has showed to reduce the incidence of early severe preeclampsia in patients with obstetric APS [47] and to improve birth rate [43,46,48].

Despite our encouraging results, we acknowledge that our study has several limitations. This is a single-center study with a low number of enrolled patients and a healthy control group whose ethnicity differed among patients. We also acknowledge that the prevalence of obesity among our healthy controls could be a bias within our data. In addition, the patients included in this study were mainly women without a known explanation for RPL. It would be interesting to test aPS/PT in a larger study that not only considers women with unexplained RPL but also patients with other identified causes in order to check for the additive effect of different variables. Along the same lines, more follow-up data and responses to treatment information would have been of interest to better address the role of aPS/PT in RPL. Further collaborative studies are needed to undertake these limitations.

## 5. Conclusions

The aPS/PT were the most prevalent aPL in healthy women with two or more consecutive miscarriages or one or more fetal deaths. Women carrying aPS/PT were mostly negative for other aPL. These antibodies acted as independent risk factors, both at the univariate and the multivariate logistic regression analysis compared to aCL and aB2GPI. All patients who had positive aPS/PT and received targeted treatment with heparin, aspirin and/or hydroxychloroquine achieved a new successful pregnancy in the follow-up.

## Figures and Tables

**Table 1:** Demographics, cardiovascular risk factors and previous treatments.

	CONTROLS (126)	EM (52)	FL (33)	EC (30)	All RPL (115)
Age and cardiovascular risk factors					
Median age (IQR)	36 (35–37)	37 (34–39)	36 (29.5–39)	36 (33–38)	37 (33–39)
Median BMI (IQR)	27.1 (24.6–31.5)	24.1 (21.5–27.5)	25.6 (23.5–28.6)	24.7 (20.8–27.3)	24.4 (21.8–27.6)
Normal weight (%)	50 (39.7%)	39 (75%)	18 (54.5%)	19 (63.3%)	76 (66.1%)
Overweight (%)	50 (39.7%)	7 (13.4%)	11 (33%)	10 (33.3%)	28 (24.3%)
Obesity (%)	26 (20.6%)	6 (11.5%)	4 (12.1%)	1 (3.3%)	11 (9.5%)
Smoke (%)	13 (10.3%)	3 (5.7%)	2 (6%)	1 (3.3%)	6 (5.2%)
Alcoholism (%)	4 (3.1%)	1 (1.9%)	0 (0%)	0 (0%)	1 (0.8%)
**Ethnicity:**					
African	3 (2.3%)	0 (0%)	2 (6%)	0 (0%)	2 (1.7%)
Arabic	9 (7.1%)	3 (5.7%)	2 (6%)	0 (0%)	5 (4.3%)
Chinese	11 (8.7%)	2 (3.8%)	1 (3%)	0 (0%)	3 (2.6%)
Caucasic	61 (48.4%)	41 (78.8%)	20 (60.6%)	27 (90%)	88 (76.5%)
Indian	2 (1.5%)	1 (1.9%)	1 (3%)	0 (0%)	2 (1.7%)
Hispanic	35 (27.7%)	5 (9.6%)	7 (21.2%)	3 (10%)	15 (13%)
Romani	5 (3.9%)	0 (0%)	0 (0%)	0 (0%)	0 (0%)
**Treatments:**					
No previous treatment	-	50 (96.1%)	33 (100%)	27 (90%)	110 (95.6%)
ASA	-	2 (3.9%)	0 (0%)	3 (10%)	5 (4.4%)
LMWH	-	2 (3.9%)	0 (0%)	3 (10%)	5 (4.4%)
HCQ	-	0 (0%)	0 (0%)	0 (0%)	0 (0%)

Cardiovascular risk factors such as age, Body Mass Index (BMI), overweight (BMI ≥ 25 and <30), obesity (BMI ≥ 30), smoking habit, alcoholism, normal weight, ethnicity and previous treatments are shown in the table above. A remarkable finding in our healthy control group is that a 60.3% of this cohort presented either overweight or obesity.

**Table 2:** Levels and prevalence of aPS/PT and criteria aPL.

**aPL Levels (U/mL)**	**Median (IQR)**				
aCL IgG	1.9 (1.9–1.9)	1.6 (1.6–1.6)	1.6 (1.6–1.6)	1.6 (1.6–1.6)	1.6 (1.6–1.6)
aCL IgM	1.9 (1.9–1.9)	1.4 (0.4–2)	0.7 (0.2–2.7)	1.4 (0.6–2.6)	0.9 (0.4–2.2)
aB2GPI IgG	1.9 (1.9–1.9)	1.4 (1.4–1.7)	1.4 (1.4–1.4)	1.4 (1.4–1.4)	1.4 (1.4–1.4)
aB2GPI IgM	1.9 (1.9–1.9)	1.4 (0.7–2.6)	0.8 (0.4–2.3)	1.4 (0.5–1.6)	1.4 (0.5–2.1)
aPS/PT IgG	7.4 (5.8–11.6)	7.5 (6.7–12.7)	7.4 (5.9–9.2)	10.7 (6.7–17.6)	7.8 (6.5–13)
aPS/PT IgM	12.3 (9.1–17.2)	19.1 (11.2–30.4)	16.4 (10.2–20.4)	14.8 (10.8–20.3)	17 (10.8–25.4)
**aPL Prevalence**	**N (%)**				
LA	0 (0%)	0 (0%)	0 (0%)	0 (0%)	0 (0%)
Any criteria aPL	0 (0%)	2 (4%)	3 (9%)	1 (3%)	5 (4.3%)
Any aPS/PT	5 (3.9%)	8 (15%)	6 (18.1%)	5 (17%)	19 (17%)
aPS/PT IgG	2 (1.6%)	4 (7.7%)	0 (0%)	4 (13.3%)	8 (6.9%)
aPS/PT IgM	3 (2.4%)	4 (7.7%)	6 (18.1%)	1 (3.3%)	11 (9.5%)

While the prevalence of the antiphospholipid antibodies (aPL) criteria in the whole cohort of patients resulted in up to 5% of all patients with recurrent pregnancy loss (RPL), positive anti-Phosphatidylserine/Prothrombin (aPS/PT) were found in 17%. Differences in aPS/PT levels were observed for aPS/PT IgM between controls and the EM group (*p* = 0.001). aCL stands for anticardiolipin. aB2GPI stands for anti-Beta-2-Glycoprotein-I.

**Table 3:** Multivariate logistic regression analysis.

	Early Miscarriage (EM) Group			Fetal Loss (FL) Group			Early Miscarriage (EM) Group		
Variable	Odds Ratio	95% CI	*p*-Value	Odds Ratio	95% CI	*p*-Value	Odds Ratio	95% CI	*p*-Value
**Obesity**	0.54	0.24–1.21	0.135	0.69	0.27–1.79	0.456	0.17	0.03–0.78	0.022
**Smoking**	0.45	0.11–1.80	0.264	0.46	0.08–2.37	0.355	0.30	0.03–2.60	0.275
**aPS/PT**	4.44	1.34–14.70	0.014	5.68	1.54–20.88	0.008	4.51	1.14–17.73	0.031

A multivariate logistic regression analysis comparing the influence of cardiovascular risk factors, such as obesity and smoking habit, in addition to aPS/PT and criteria aPL, was performed to weigh up the role of aPS/PT found in the univariate study. Smoking habit, obesity and criteria aPL did not posed a significant risk for any of the forms of RPL studied. In contrast, aPS/PT showed a contribution in risk of RPL in all groups. Obesity resulted in a protective factor for the EC group, probably lead by the high prevalence of this particular cardiovascular risk factor among our healthy control cohort.

**Table 4:** Obstetrical history, aPL levels, treatment and outcome of patients carrying positive aPL.

Patient No	aPS/PT IgG	aPS/PT IgM	aCL IgG	aCL IgM	aB2GPI IgG	aB2GPI IgM	LA—dRVVT Ratio	LA—SCT Ratio	ANA	ANTI-DNA	Age	P	M	PW of PL before Treatment	Treatment	LMWH	ASA	HCQ	Outcome
**EM1**	**30.6**	12.2	1.6	0.2	1.4	0.9	1.03	0.98	N	N	34	4	3	6, 9, 6	Yes	Yes	Yes	Yes	Pregnancy and HN
**EM2**	**47.3**	23.3	1.6	4.1	1.5	5	0.99	1.08	N	N	40	4	3	6, 6, 8	Yes	Yes	Yes	No	Pregnancy and HN
**EM3**	**42.1**	15.2	1.6	0.7	1.4	0.6	1.14	1.17	N	N	39	5	4	VIP, 6, 5, 5, 6	Yes	Yes	Yes	No	Pregnancy and HN
**EM4**	13.5	**40**	1.6	0.2	2.18	1.4	0.84	1	N	N	35	4	4	9, 7, 9					
**EM5**	6.64	**48**	1.6	1.5	1.4	2.7	0.81	1.16	N	N	31	3	3	6, 8, 7					
**EM6**	**33.2**	23.4	1.6	5.8	1.4	5.6	0.96	1	N	N	39	4	4	8, 8, 6, 7					
**EM7**	12.5	**59.5**	1.6	0.2	1.4	0.3	0.98	0.94	N	N	40	4	3	Delivery, 6, 8, 9, 5					
**EM8**	6.06	**42.5**	1.6	2.4	1.4	2.8	0.95	0.93	N	N		4	4	6, 6, 9, 8					
**EM9**	8.05	38.2	14.5	**52.4**	12.6	**56**	1.03	1.09	N	N	35	6	6	6 PL < PW10	Yes	Yes	Yes	Yes	Miscarriage at PW 8
**FL1**	21.8	**57.7**	9.6	7.8	8.5	6.8	1.02	0.95	N	N	38	4	2	11, 13, 30	Yes	Yes	Yes	No	Pregnancy and HN
**FL2**	7.87	**53.3**	1.6	2.5	1.4	2.2	0.85	0.82	N	N	33	2	2	20, 40	Yes	Yes	Yes	No	Pregnancy and HN
**FL3**	11.3	**71.6**	1.6	0.5	1.4	0.5	1.13	1.27	N	N	31	5	4	12, 11, 12, 12					
**FL4**	21.6	**64.3**	**73.7**	**22.2**	**87.7**	**23.2**	1.05	1.26	N	N	41	3	3	18, 18, 18					
**FL5**	7.1	**65.1**	1.6	1.5	1.4	3.1	1.05	1.17	N	N	30	2	1	29 (Placental thrombi)					
**FL6**	7.47	**74.8**	1.6	8.6	1.4	8.6	0.99	1.13	N	N	38	5	3	12, 18, 15					
**FL7**	19	8.57	2.1	4.9	**29.3**	1.4	0.96	0.82	N	N	40	3	3	11, 11, 14	Yes	Yes	No	No	Pregnancy and HN
**FL8**	5.25	8.59	3.1	**23.6**	1.4	1.6	1	1.09	N	N	25	3	1	VIP, 16	Yes	Yes	No	No	Pregnancy and HN
**EC1**	**40.1**	20.3	1.6	0.2	1.4	0.2	0.87	0.96	N	N	32	2	2	7, 5	Yes	Yes	Yes	No	Pregnancy and HN
**EC2**	**45.2**	11.6	1.6	2	1.4	1.9	1.06	0.97	N	N	44	2	2	8, 6	Yes	Yes	Yes	Yes	Pregnancy and HN
**EC3**	**53.5**	33.8	1.6	1.6	1.4	2.5	1	1.1	N	N	38	2	2	8, 5	Yes	Yes	Yes	Yes	Pregnancy and HN
**EC4**	5.87	**52.7**	1.6	0.6	1.4	0.5	0.89	1.17	N	N	35	2	2	9, 6					
**EC5**	**206**	10.8	1.6	1.8	3.07	1.4	0.95	1.23	N	N	43	2	2	7, 8					
**EC6**	9.05	9.47	2.5	**47.1**	1.4	6.8	0.97	1.19	N	N	34	4	2	2 Deliveries, 9, 6	Yes	Yes	Yes	No	Pregnancy and HN

Rows start with the first column identifying the case number within each of the patients’ groups (Early Miscarriage or EM, Fetal Loss or FL and Extra-Criteria or EC). Bold values in the first column indicate positive aPS/PT cases. All of them who received targeted treatment achieved a new pregnancy, with delivery of an alive newborn. All patients resulted negative in LA (dRVVT ratio and SCT ratio), ANA and anti-dsDNA testing. P stands for the number of pregnancies, M stands for the number of miscarriages, PW stands for pregnancy week, VIP stands for voluntary interruption of pregnancy, PL stands for pregnancy loss and HN stands for healthy newborn.

## Data Availability

Data included in the article have been generated within our hospital and are included in this article. Further data available on request.
